# Enhanced salience of edge frequencies in auditory pattern recognition

**DOI:** 10.3758/s13414-024-02971-x

**Published:** 2024-10-26

**Authors:** Michel Bürgel, Diana Mares, Kai Siedenburg

**Affiliations:** 1 Dept. of Medical Physics and Acoustics, Carl Von Ossietzy University of Oldenburg, 26129 Oldenburg, Germany; 2 Signal Processing and Speech Communication Laboratory, Graz University of Technology, 8010 Graz, Austria

**Keywords:** Auditory attention, Auditory scene analysis, High voice superiority effect, Music perception, Salience

## Abstract

Within musical scenes or textures, sounds from certain instruments capture attention more prominently than others, hinting at biases in the perception of multisource mixtures. Besides musical factors, these effects might be related to frequency biases in auditory perception. Using an auditory pattern-recognition task, we studied the existence of such frequency biases. Mixtures of pure tone melodies were presented in six frequency bands. Listeners were instructed to assess whether the target melody was part of the mixture or not, with the target melody presented either before or after the mixture. In Experiment 1, the mixture always contained melodies in five out of the six bands. In Experiment 2, the mixture contained three bands that stemmed from the lower or the higher part of the range. As expected, Experiments 1 and 2 both highlighted strong effects of presentation order, with higher accuracies for the target presented before the mixture. Notably, Experiment 1 showed that edge frequencies yielded superior accuracies compared with center frequencies. Experiment 2 corroborated this finding by yielding enhanced accuracies for edge frequencies irrespective of the absolute frequency region. Our results highlight the salience of sound elements located at spectral edges within complex musical scenes. Overall, this implies that neither the high voice superiority effect nor the insensitivity to bass instruments observed by previous research can be explained by absolute frequency biases in auditory perception.

**Supplementary information:**

The online version contains supplementary material available at 10.3758/s13414-024-02971-x.

## Introduction

Most acoustic scenes in the real world comprise sounds from multiple sources. Consider the realm of music, wherein the experience of listening to compositions featuring multiple instruments playing simultaneously is commonplace. In these complex scenes, certain instruments and their sounds and melodies often seem more prominent, standing out amidst the complex texture of overlapping sounds. Think of sung melodies, the lines of wind instruments, or guitar riffs—these elements frequently capture our attention. By contrast, the melodies of bass instruments less frequently stand out. This might suggest a potential perceptual bias that inhibits the recognition of low-frequency sound elements or melodies. Here, we sought to study such spectral biases in auditory scene analysis (ASA; Bregman & McAdams, 1994).

Principles of ASA are key to understanding how the human auditory system differentiates sounds within complex auditory scenes. ASA encapsulates a range of processes through which the auditory system organizes sound elements by segregation and grouping to craft coherent mental representations known as streams, following Gestalt principles. This is achieved by utilizing external sound features (bottom-up) and internal cognitive processes of the listener (top-down).

When dissecting a musical scene, bottom-up processing entails recognizing spectrally distinct sounds, differences in their continuity (onset and offset), or variations in spectrum. These factors contribute to the interpretation of distinct instruments and melodies within the scene. Conversely, top-down processing relies on learned musical patterns, expectations, and familiarity with specific instrumentations or musical arrangements, aiding in scene parsing.

The role of auditory attention is the topic of an ongoing discussion (for a review, see Snyder et al., 2012; Sussman, 2017). Despite discrepancies regarding the extent to which attention affects ASA, studies indicate that attention facilitates the organization of sounds (Alain & Arnott, 2000; Sussmann, 2006) and that it can emphasize otherwise hidden elements in auditory scenes (Sussman & Steinschneider, 2009). Investigating the audibility of instruments within musical scenes, Bürgel et al. (2021) conducted an instrument-detection experiment using popular music excerpts, building upon a paradigm by Bey and McAdams (2002). Participants were tasked with detecting target instruments embedded in excerpts of popular music featuring mixtures of instruments. Target sounds included various instrument categories, such as bass instruments and singing voices. In half of the mixtures, the target was absent. To cue participants to the target, an isolated target track identical to the one embedded in the mixture was presented. The study varied whether the target cue was presented first or after the mixture: In cases where the target was played first, participants could use prior knowledge gained from the cue to direct attention towards the target in the mixture, making detection dependent on both its acoustical salience and selective attention. When the mixture was presented first, no cue was given, and performance relied much more on the salience of the target. The comparison of presentation orders allowed us to isolate the impact of selective attention from the target’s inherent acoustical salience, revealing the specific influence of attentional gain.

Results indicated that the presentation order considerably impacted detection, with superior detection observed when the target was presented first. Notably, the extent of this impact varied across instrument categories, with vocal sounds exhibiting almost no effect and bass instruments displaying the most significant decrease in detection accuracy among all studied sounds. This suggested that when attention was not directed towards the bass, it was less likely perceived in the musical scene, suggesting the notion of a spectral hierarchy. This diminished accuracy for bass sounds persisted even when the sounds were aligned in sound level. One interpretation of these findings is that the human auditory system exhibits spectral biases, which have the greatest influence when freely listening in to an auditory scene.

Studies investigating polyphonic music with multiple independent voices have consistently reported a high-voice superiority effect (HVSE; Crawley et al., 2002), which could be related to potential spectral biases. The HVSE refers to the phenomenon where, in the context of multiple simultaneous melodies, the melody with the highest pitch trajectory is more prominent in the cortical responses of listeners, rendering it more salient in polyphonic scenes (Fujioka et al., 2005). This effect has been observed even in infants (Marie & Trainor, 2013) and may stem from physiological factors within the human auditory system, influenced by the interplay of harmonic structures in tone complexes (Trainor et al., 2014). Research indicates that musical training in instruments within the soprano range enhances this effect, whereas training in the bass range does not reverse the effect but can lead to an equalization between lower and higher voices (Marie et al., 2012). Moreover, studies on instruments in the bass range have reported that while bass instruments may yield challenges in melody perception, they exhibit superior time perception (Hove et al., 2014). Taken together, these findings appear to imply that the human auditory system possesses mechanisms favoring melodies of higher voices in musical scenes.

Given this background, we explored whether ASA is subject to spectral biases, in which sounds within specific frequency regions possess a distinct salience in auditory pattern matching tasks. Specifically, we aimed to discern absolute and relative biases. Absolute biases would manifest as increased salience of bands in distinct frequency regions, whereas relative biases would manifest systematically at specific positions within the musical scene regardless of the absolute position in the auditory spectrum. To study this, the same task as in Bürgel et al. (2021) was used with acoustically more controlled stimuli where natural musical instruments were replaced by random pure tone melodies in different frequency bands.

We hypothesized that the presentation order between the target cue and mixture, as well as the frequency regions in which the target melodies appear, would impact detection accuracy. Specifically, due to the observed diminished performance of bass instruments, we expected the melodies in the lower frequencies to exhibit the poorest performance, with a significantly larger decline between the target-first and mixture-first presentation orders. Conversely, due to the reported HVSE, we expected the melody in the highest frequency band to be more salient and outperform melodies in the low- and mid-frequency region. Additionally, we assumed that the HVSE would lead to better performance for melodies in high frequency bands, highlighting biases towards specific absolute frequency regions.

## Experiment 1

### Methods

#### Participants

A total of 26 typical-hearing participants took part in the experiment. While formal power analyses were not conducted, the sample size was based on previous studies employing a similar detection paradigm (Bürgel et al., 2021, order effect—Experiment 1: β =  − 0.710, *t* = 15.542, *p* < 0.001; Bürgel & Siedenburg, 2023, order effect—Experiment 1: β =  − 0.545, *t* = 9.356, *p* < 0.001). One participant was excluded due to obtaining a below-chance performance in the Target-Mixture condition. Of the remaining 25 subjects (mean age = 26.5 years, *SD* = 5.23; age range: 19–38, four diverse, seven women, 14 men), 20 were categorized as musicians. In our study, musicianship was assigned based on whether an individual had received 3 or more years of formal training on a musical instrument, including voice. Additionally, participants’ musical abilities were evaluated using questions from subscales of the Goldsmith Musical Sophistication Index (Gold-MSI; Müllensiefen et al., 2014). The average Gold-MSI score for the Perceptual Abilities subscale was 53.0 (*SD* = 8.52) for musicians and 39.8 (*SD* = 11.26) for nonmusicians, and the one for the Musical Training subscale was 35.85 (*SD* = 5.45) for musicians and 15.8 (*SD* = 4.38) for nonmusicians.

#### Stimuli

**Fig. 1:**
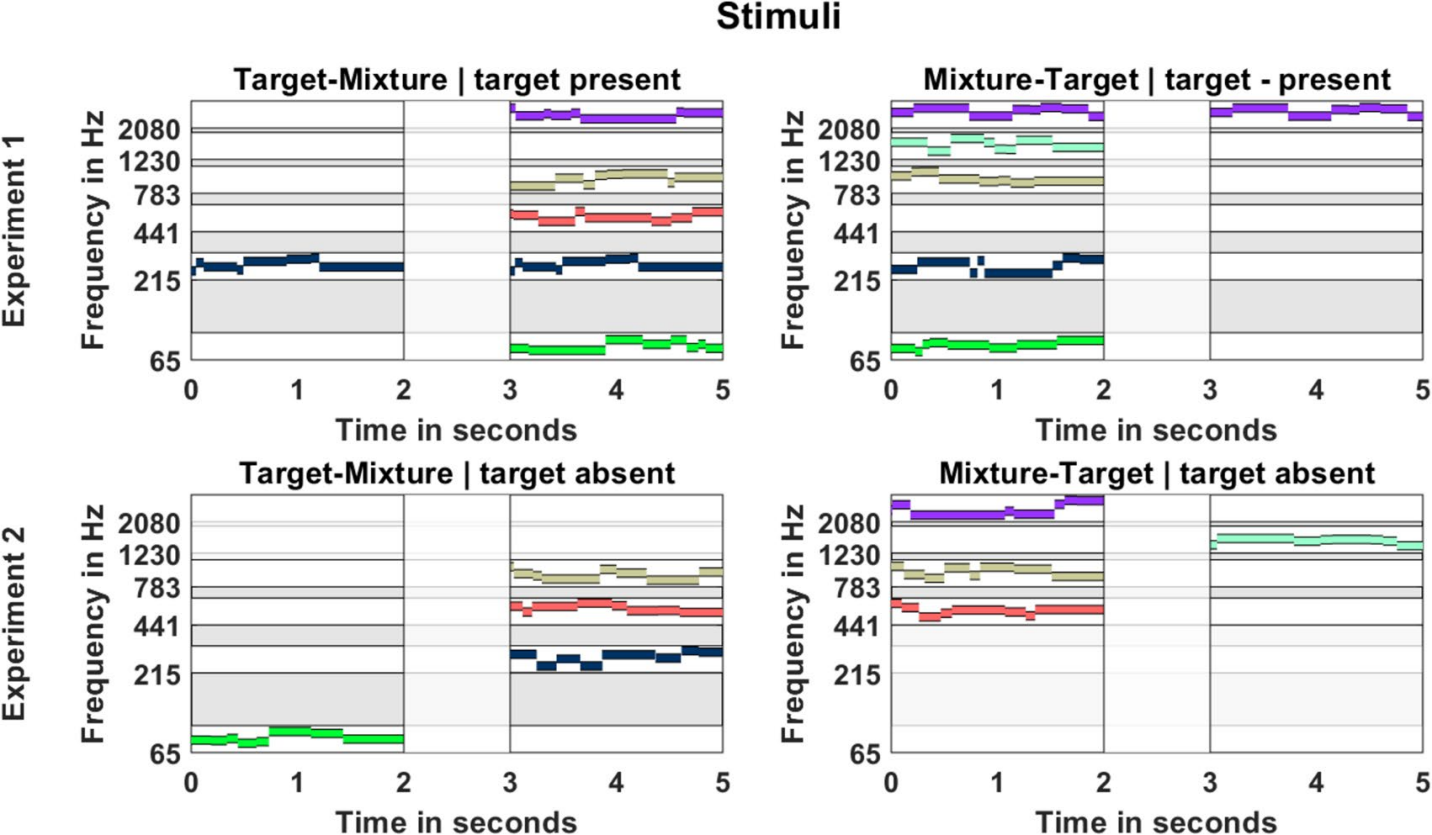
Illustration of the stimuli: Isolated 2-s target melodies were presented either before a 2-s mixture (Target-Mixture) or afterwards (Mixture-Target), with a 1-s pause in between. The target could be present or absent from the mixture. In Experiment 1, five melodies played in the mixture occurring in separated frequency bands. In Experiment 2, the frequency bands were split into a low and high range, each containing four frequency bands, and only three melodies were playing simultaneously. The colored lines represent example melodies in each band. The gray area represents the empty space where no melody could occur. (Color figure online)

A schematic of the stimuli is presented in Fig. 1. To mitigate energetic masking, each melody occupied a designated frequency space, maintaining sufficient frequency distances between melodies. Six frequency bands were used in the experiments, which were spaced on the equivalent rectangular bandwidth (ERB) scale (Glasberg & Moore, 1990) with center frequencies between 65 and 2080 Hz. The target was present in half of the trials. Because the absence of the target resulted in a less dense mixture, one random band was muted each time the target was present in the mixture.

The stimuli were created on the fly (open set design). Each frequency band contained eight pseudorandomized pure-tone melodies, each lasting 2 s. Tone frequencies were drawn randomly from a uniform distribution on a logarithmic frequency scale with half-octave range, anchored at the center frequencies of the six ERB bands (65, 215, 441, 783, 1300, 2080 Hz). Frequencies were drawn with no constraints applied to musical intervals or semitone steps. For instance, melodies in the 1300-Hz band could encompass notes ranging from 1300 to 1838 Hz (1300 Hz + ½ octave). This approach ensured that frequencies spanned a sufficient frequency range to be discernible as melodies while maintaining sufficient spacing between bands. The resulting frequency distance between neighboring tones was at least one ERB, to mitigate the potential effects of energetic masking. Tones had random durations, with onset and offset timepoints of the eight-tone sequence generated by drawing seven timepoints from a uniform distribution between 0 and 2. These randomly drawn timepoints were sorted in ascending order, and drawings were discarded that contained durations shorter or equal than 50 ms. Timepoint *n* would here serve as offset of tone *n* and onset of tone *n* + 1; the onset of the first tone of the sequence was defined as *t* = 0 ms and the offset of the last as *t* = 2,000 ms. Tones were then synthesized with the defined onset/offset times and separated by 10 ms offset and 10 ms onset cosine ramps.

To eliminate sound level cues all sounds were aligned in level using A-weighting and every band was presented at a level of 40 dB SPL (A). In the initial section of the experiment, the target was followed by the mixture (Target-Mixture condition), while in the final section, the order was reversed (Mixture-Target condition). The frequency conditions were randomized.

#### Procedure

First, participants were informed about the experiment and provided informed consent. A short training session followed, during which participants received feedback. Afterwards, the main experiment started, and no more feedback was provided. After the completion of the main experiment, each participant filled out a brief questionnaire comprising demographic data and components of the Gold-MSI. Participants were financially compensated.

Experiment 1 was divided into two blocks: Target-Mixture and Mixture-Target. Each block contained all six frequency conditions. The structure of a trial was as follows: presentation of a target sequence (2 s), short pause (1 s), presentation of a mixture (2 s). The second block maintained this structure but interchanged the presentation of the target with the one of the mixture. In both instances, the task was to detect whether the target was present in the mixture (yes/no task).

The Target-Mixture block comprised a total of 120 trials, with 20 trials allocated for each frequency condition. Following a short break, the experiment proceeded with the Mixture-Target block, throughout which 240 trials, with 40 trials per frequency condition, were presented. This second section included a break after the first 120 trials. In both blocks, the number of instances in which the target was present or absent from the mixture was counterbalanced between frequency conditions. The different number of stimuli between two order conditions was due to experience from pilot experiments: It was anticipated that detection performance would approach ceiling level for many participants in the Target-Mixture condition, while performance in the Mixture-Target condition was expected to be lower. Therefore, for the sake of precise performance estimates, we increased the number of stimuli in the Mixture-Target condition while the Target-Mixture condition acted as a control condition.

In the initial version of the experiment the mixture comprised six simultaneous melodies (one in each frequency band) when the target was present and five when the target was absent. There was a concern that participants might adopt a strategy of counting the number of melodies instead of focusing on detecting the target melody. To prevent this potential strategy, a second version of the experiment was conducted. In this version, when the target was present, one random nontarget melody frequency band was muted. This adjustment ensured a consistent density of five melodies in the mixture, regardless of the target’s presence. As there were no statistically significant differences found between the two versions, with a correlation of *R*^2^ = 0.94 between the mean results of both variants of the experiment, the results of all participants were analyzed conjointly.

#### Apparatus

The experiment took place in a double-walled sound booth at the University of Oldenburg. Participants sat in a comfortable chair and interacted with the experiment using a touch screen attached to a movable arm in front of them. Stimuli were processed through an RME Fireface UCX soundcard at a 44.1 kHz sampling rate and presented on Sennheiser HD 650 headphones. Stimuli were synthesized in MATLAB. Sound levels were measured with a Brüel & Kjær Type 2250 light sound-level meter and a Brüel & Kjær Type 4153 artificial ear, to which the headphones were coupled.

### Results and discussion

**Fig. 2:**
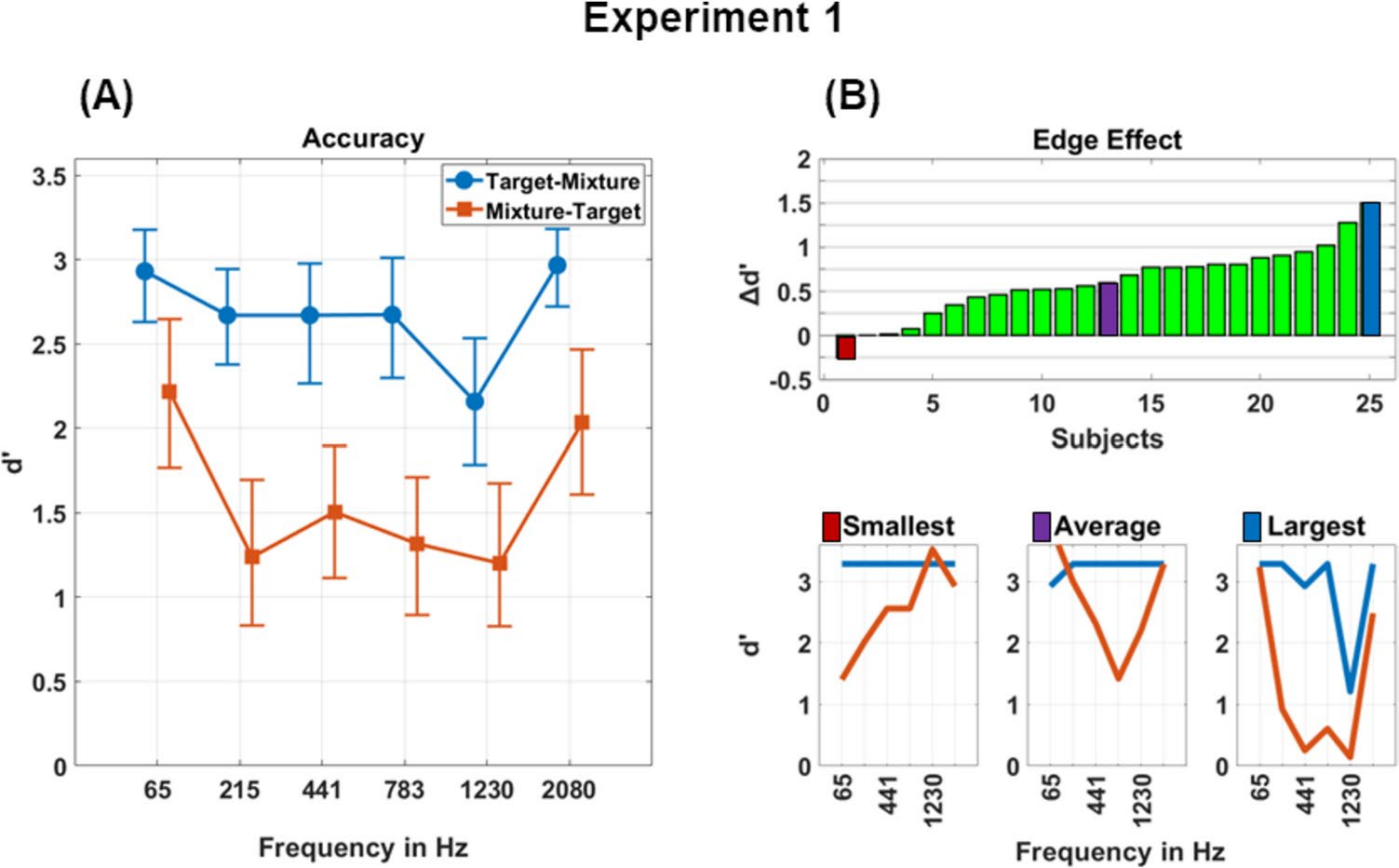
**(A)** Detection accuracy in Experiment 1 is represented as *d′* scores. The square and circle marks denote the mean scores for melodies in the specified frequency bands. The square marks indicate the presentation order Target-Mixture, where the target cue was presented first followed by a mixture. The circle marks indicate the presentation order Mixture-Target, where a mixture was presented first followed by the target cue. Error bars represent 95% CIs computed using a bootstrapping method. **(B)** For each subject the magnitude of the edge effect is displayed as the difference between mean *d′* scores for melodies in the first and last frequency band and mean *d′* scores for melodies between the first and last frequency band. The bottom figures display individual *d′* scores for the subject with the smallest, the average, and the largest edge effect, respectively. (Color figure online)

The average performance in Experiment 1 is displayed as *d*-prime (*d′*) scores in Fig. 2A (numerical values are available in Supplementary Table 1). When comparing the presentation orders by averaging over frequency conditions, the Target-Mixture condition yielded clearly better detection scores, with an average score of *d′* = 2.68, compared with the Mixture-Target condition with an average score of *d′* = 1.58 (− 1.10).

Differences between melodies at the edge of the frequency range (lowest and highest) and those in the middle revealed an edge effect, with edge frequency bands yielding an average score of *d′* = 2.54 compared with mid frequencies with an average score of *d′* = 1.93 (− 0.61). Edge effects were pronounced in both presentation orders, with scores for edges in the Target-Mixture condition of *d′* = 2.95, compared with scores for mid frequencies of *d′* = 2.55 (− 0.40), and for edges in the Mixture-Target condition with a score of *d′* = 2.12, compared with scores for mid frequencies of *d′* = 1.31 (− 0.81). To further evaluate the observed effects, the difference between the performance of edge and middle frequencies was computed for each participant separately. The magnitude of individual edge effects is displayed in Fig. 2B. Four participants showed differences close to zero (*d′* < 0.1), wherein one participant exhibited better performance for the center frequencies with a reversed edge effect of *d′* =  − 0.26. Overall, however, the majority of participants demonstrated clear edge effects.

A linear mixed-effects model (LME) was employed to analyze the data, incorporating random intercepts for each participant. Presentation order and categorization of whether targets appeared as edge frequencies versus in middle frequencies were used as binary predictors. Musical sophistication scores and the frequency band in which the melody appeared were used as numerical predictors. The factors presentation order and edge frequency showed pronounced effects as well as an interaction (order: β = 0.514, *t* = 13.479, *p* < 0.001; edge: β = 0.303, *t* = 7.947, *p* < 0.001; interaction: β = 0.106, *t* = 2.765, *p* = 0.005). The effects of frequency band and musical sophistication were negligible (frequency band: β = 0.042, *t* = 0.304, *p* = 0.761; musical perception: β = 0.004, *t* = 0.268, *p* = 0.788; musical training: β = 0.074, *t* = 0.401, *p* = 0.522). Further underlining the lack of impact of musical sophistication, correlations between participants’ averaged *d′* scores and sophistication scores revealed *R*^2^ values below 0.05 for both musical perception and musical training.

To further investigate the effect of local spectral edges caused by muting frequency bands adjacent to the target, we compared the results of detecting targets where a band adjacent to the target band was omitted and melodies where a band not adjacent to the target was omitted (see Supplementary Fig. 1). The results showed a close alignment in detection performance between the two conditions, with no considerable differences observed in the LME (main effect: β =  − 0.064, *t* =  − 0.169, *p* = 0.866; interaction with presentation order: β =  − 0.167, *t* =  − 0.699, *p* = 0.485). These findings suggest that there was no discernable impact of local edges resulting from the removal of aligned frequency bands. Additionally, the absence of differences between both conditions can be interpreted as an indication that energetic masking did not substantially contribute to the detection process. If energetic masking had a substantial effect, conditions with a missing neighbor would have performed better than those with neighbors present on both sides.

Taken together, the observed order effect corroborates previous reports on the facilitating role of attention in ASA (Alain & Arnott, 2000; Bey & McAdams, 2002; Bürgel et al., 2021; Woods & McDermott, 2015). Prior knowledge could be used to direct auditory attention towards the target sound and thus follow and highlight auditory representations in the auditory scene. Contrary to our hypothesis, neither a general bias towards higher frequencies, nor an unawareness of activity in lower frequencies was observed. Instead, the results revealed a superior detection for both edges of the auditory scene. These results raise the question of whether an effect of relative frequency bias was at play, wherein the spectral edges of the acoustic scene were better recognized compared to sounds located closer to the frequency center of the scene, or whether the resulting pattern was due to an absolute frequency bias in auditory pattern matching.

## Experiment 2

In Experiment 2, we aimed to explore whether the previously observed edge effect persisted despite global changes in frequency. For this purpose, the frequency range was divided into two equal ranges. The first range comprised Frequency Bands 1–4, and the second range comprised Frequency Bands 3–6.

### Method

#### Participants

Thirty typical-hearing participants with a mean age of 24.7 years (*SD* = 2.54, range: 20–29; two diverse, 17 women, 11 men) performed the second experiment. Following the predefined criterion, 14 were designated as musicians. No participant was excluded. The average Gold-MSI score for the Perceptual Abilities subscale was 53.07 (*SD* = 7.26) for musicians and 44.31 (*SD* = 7.11) for nonmusicians and the one for the Musical Training subscale was 32.42 (*SD* = 5.97) for musicians and 13.37 (*SD* = 6.67) for nonmusicians.

#### Stimuli and procedure

Experiment 2 consisted of four blocks: two Target-Mixture blocks and two Mixture-Target blocks. One block of each order condition contained conditions in the lower frequency range (Bands 1–4) while the other block contained the ones in the higher range (Bands 3–6). The order of the frequency ranges was counterbalanced, such that each participant began either with the low or the high range in the Target-Mixture block. Each condition was presented 20 times in each Target-Mixture block, which summed up to a total of 160 trials for the first two blocks. The Mixture-Target blocks comprised twice the number of trials compared with the Target-Mixture block, with a total of 320 trials. After each block, a short break followed. The mixture density was constrained to three simultaneous frequency bands across all trial types. The training section involved eight Target-Mixture trials—four in the lower frequency range and four in the higher frequency range.

### Results and discussion

**Fig. 3:**
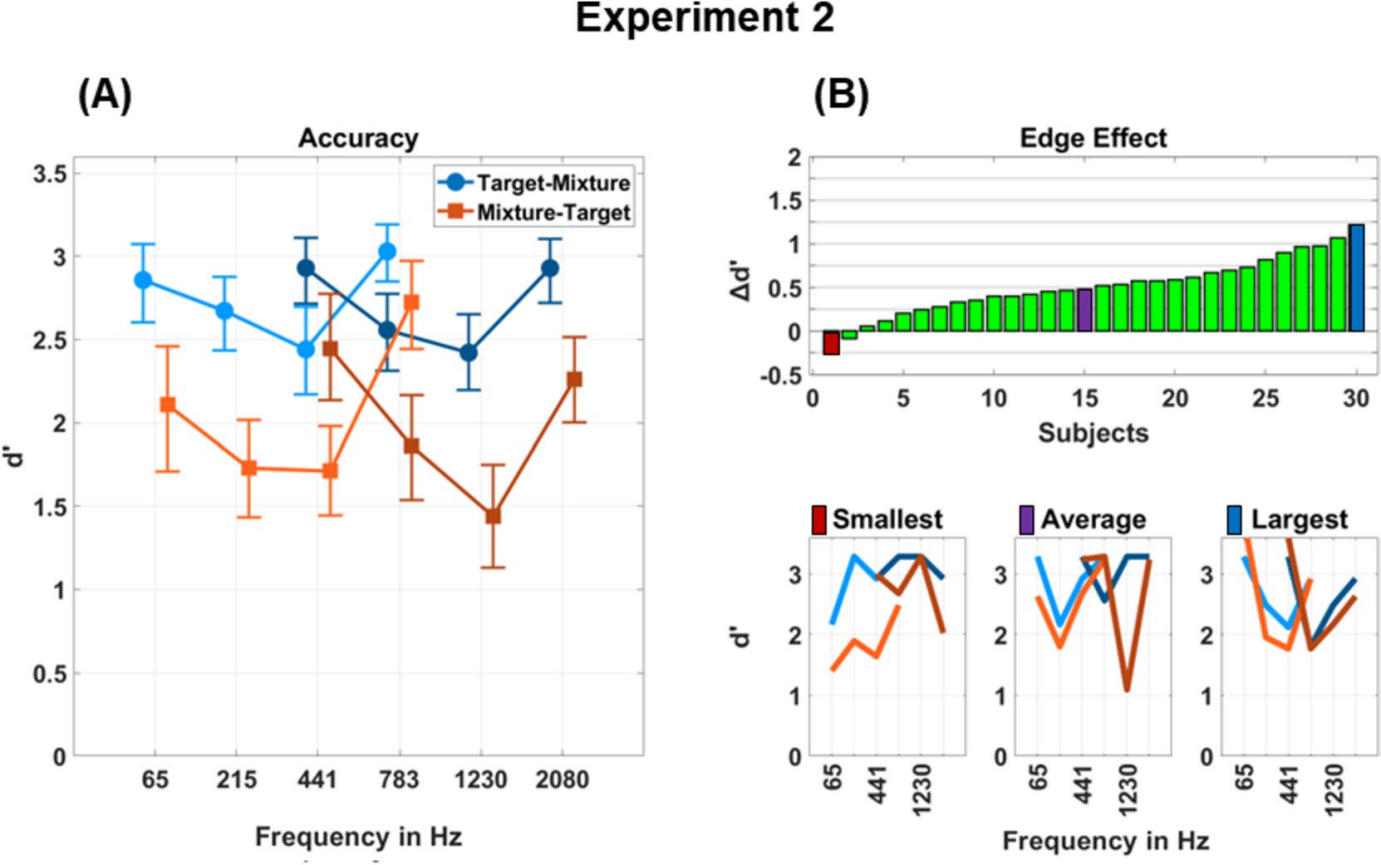
**(A)** Detection accuracy in Experiment 2 is represented as *d′* scores. Three melodies were presented simultaneously within either a low- or high-frequency range. The brighter color indicates the low-frequency range (65–783 Hz), and the darker color represents the high-frequency range (441–2080 Hz). The square and circle marks denote the mean scores for target melodies in the specified frequency bands. The square marks indicate the presentation order Target-Mixture, where the target cue was presented first followed by a mixture. The circle marks indicate the presentation order Mixture-Target, where a mixture was presented first followed by the target cue. Error bars indicate 95% CIs computed using a bootstrapping method. **(B)** For each subject the magnitude of the edge effect is displayed as the difference between mean *d′* scores for melodies in the first and last frequency bands and mean *d′* scores for melodies in the second and third frequency bands. The bottom figures display individual *d′* scores for the subject with the smallest, the average, and the largest edge effect, respectively. (Color figure online)

Results are displayed in Fig. 3A (numerical values are available in Supplementary Table 2). Similar to Experiment 1, when examining the difference between presentation orders by averaging over frequency and frequency range conditions, the Target-Mixture condition showed clearly higher scores (*d′* = 2.75), compared with the Mixture-Target condition (*d′* = 2.07).

When examining the averages within each frequency range, the low-frequency range exhibited a slightly better score of *d′* = 2.41 compared with the high-frequency range with a score of *d′* = 2.36 (− 0.05). Differences between frequency conditions at the edge of the frequency ranges and those in the center of the ranges revealed an edge effect, with edge frequencies achieving better detection with an average score of *d′* = 2.66 compared with mid frequencies with an average score of *d′* = 2.10 (− 0.56). Negligible differences within the edge effects between presentation orders or intervals were observed, with scores for the Target-Mixture condition in low and high ranges both being *d′* = 2.93, and for the Mixture-Target condition in low and high ranges being *d′* = 2.42 and *d′* = 2.36, respectively. To further evaluate the observed effects, the difference between the performance of edge and middle frequencies was computed for each participant separately. The magnitude of individual edge effects is displayed in Fig. 3B. Three participants showed differences close to zero (*d′* < 0.1), whereas two participant exhibited better performance for the center frequencies with reversed edge effects of Δ *d′* =  − 0.26 and Δ *d′* =  − 0.09. Overall, the majority of participants demonstrated an edge effect, whereby the most pronounced effect showed an enhancement of Δ *d′* = 1.22.

Participants with higher musical sophistication scores showed better detection accuracy. Correlations based on musical perception scores yielded an *R*^2^ of 0.16, while those based on musical training scores yielded an *R*^2^ of 0.36. Differences between order conditions were apparent, with an *R*^2^ of 0.06 for perception and an *R*^2^ of 0.18 for training in the Target-Mixture condition.

Larger correlations were observed in the Mixture-Target condition, reaching an *R*^2^ of 0.21 for perception and an *R*^2^ of 0.43 for training. The LME used in Experiment 2 utilized the same fixed effects as in Experiment 1, with the addition of a binary variable determining whether the stimulus appeared in the low- or high-frequency range. Effects were pronounced for presentation order and whether frequencies appeared on the edges of a frequency range, as well as for musical training scores (order: β = 0.353, *t* = 13.064, *p* < 0.001; edge: β = 0.267, *t* = 9.790, *p* < 0.001; musical training: β = 0.028, *t* = 3.084, *p* = 0.003; frequency band: β = 0.031, *t* = 1.164, *p* = 0.245; frequency range: β = 0.023, *t* = 0.767, *p* = 0.443; musical perception: β = 0.015, *t* = 1.226, *p* = 0.297).

As a consequence of the experimental design, there were trials where the melody in the first or last frequency band was muted, resulting in the target melody in the second or third band taking the role of an edge frequency. For example, if Frequency Band 1 was muted, Band 2 became the lowest frequency in the mixture. In the reported results above, only instances where target melodies were truly embedded in the center of the musical scene, with melodies in frequency bands above and below the target frequency band, were analyzed. To investigate how the relative position of frequency bands impacted the detection of melodies, a separate analysis was conducted. This involved analyzing separate hit rates for both variants using a generalized linear-effects model to account for the constrained nature of hit rates. The model employed the same effects as the LME model, except hit rates were used as a response variable. As observed for frequency bands on the edge of the frequency range, an edge effect was evident even within the same frequency band (*F* = 13.059, *p* < 0.001). Precisely, melodies on the edge outperformed melodies in the center, achieving hit rates of 89 [0.82–0.94] percentage points compared with the 75 [0.66–0.83] percentage points achieved by melodies in the center (see Supplementary Fig. 2).

Overall, Experiment 2 revealed pronounced edge effects across different frequency regions. Furthermore, a weak positive effect of musical training was evident, suggesting that individuals with higher musical sophistication scores also have improved abilities for the detection of melodies in complex acoustical scenes. The absence of this impact observed in Experiment 1 may be attributed to the participant pool, primarily consisting of musicians with a small range of musical sophistication scores.

## General discussion

In line with our hypotheses and consistent with previous research (Bey & McAdams, 2002; Bürgel et al., 2021), the presentation order of the target played a crucial role in target detection, highlighting the influence of top-down processing on ASA. Presented with the target before the mixture, listeners were able to leverage this information to selectively direct attention towards the melody in the target frequency band, which resulted in a higher detection accuracy compared with the order where the target was presented after the mixture. Our hypothesis regarding perceptual biases towards specific absolute frequency regions, on the other hand, was clearly refuted. Instead of an inferior detection of low-frequency bands or superior detection of high-frequency bands, detection was facilitated for frequencies that appeared on the edges of the acoustical scene, implying biases towards relative frequency regions. This edge effect was consistent for melodies on the relative outer frequency bands of the acoustical scene, regardless of absolute frequency. The effect was even more pronounced in the more difficult Mixture-Target condition. We interpret this effect as a salience phenomenon, where the melodies at the spectral edges attract auditory attention, thereby detracting from melodies in between. This effect particularly shapes perception when listening to the scene holistically without prior target (Mixture-Target). In line with these findings, participants informally stated that they had used the outer melodies as landmarks to subsequently hear out melodies between these. It would be interesting to explore whether the occurrence of edge effects is influenced by acoustic cues within the melodies, such as tone onsets and frequency trajectories, or if the edge effects can be fully attributed to the frequency bands. This could be investigated by examining whether such effects persist even when the melodies are replaced with static tones within the individual frequency bands.

MSI scores had a positive effect on melody detection (Müllensiefen et al., 2014). While this effect was not observed in the studies by Bey and McAdams (2002) and Bürgel et al. (2021), it aligns with numerous reports in the literature where musical training scores are positively associated with performance in music related tasks. It is reported that individuals with higher scores exhibited better perceptual abilities, such as pitch and rhythm discrimination (Kannyo & DeLong, 2011; Marozeau et al., 2010; Micheyl et al., 2006; Tervaniemi et al., 2005), as well as the ability to recognize melodies and instruments in musical scenes (Crawley et al., 2002; Siedenburg et al., 2020; Slater & Marozeau, 2016). Musicians’ auditory skills may have enabled them to better isolate individual melodies, allowing them to search the scene for the target melody more efficiently. Nevertheless, better recognition by musically trained individuals did not compensate for the order effect or the edge effects, indicating that these phenomena are fundamental even for high-performing individuals.

The enhanced detection of edges, where no difference was observed between the lower and upper edges, may initially appear at odds with the HVSE (Fujioka et al., 2005; Hove et al., 2014; Marie et al., 2012; Marie & Trainor, 2013, 2014). However, studies on the HVSE suggest that it is grounded in the nonlinear processing of harmonics in tone complexes, where the harmonics of the higher voice are more likely to mask those of the lower voice (Trainor et al., 2014). In our experiment, the use of pure tones without harmonic overtone structures thus may account for the absence of this perceptual hierarchy. Comparing this study with HVSE studies suggests additional discrepancies. HVSE studies typically involve only two simultaneous melodies, which could be interpreted as a comparison between the lower and upper edges, which exhibited no significant differences in our experiment. However, such an interpretation would disregard the potential influence of other melodies in the center of the presented ranges. Furthermore, the edge effect stands in striking contrast to our findings using acoustic excerpts of pop music that comprised sounds from real musical instruments, wherein bass instruments had the most pronounced detection differences between both presentation orders and also the lowest detection score among tested instruments (Bürgel et al., 2021), even though the sound levels of the instruments were equalized. The present edge effect, however, challenges the notion that the diminished performance of bass instruments is solely due to spectral biases (whether absolute or relative). Several factors may contribute to these discrepancies. Unlike the pure tones used in our experiment, sounds of bass instruments comprise tone complexes that spectrally overlap with other instruments in the musical scene. This overlap might result in the lower voice being more easily masked by the higher ones, as explained by HVSE. Importantly, in contrast to the randomly generated melodies in our experiment, which had no systematic relationship to each other, instruments and their lines or melodies in musical compositions are often created in a deliberate relationship to each other, both in pitch and in time. Bass instruments, in particular, often provide the harmonic basis of tonal music, thus support other melodies besides providing rhythmic accents, which associates them more with rhythm and groove perception (Hove et al., 2014). However, these musical properties also make bass instruments more likely to be part of the musical background and avoid standing out.

The results obtained in our two experiments are concordant with research investigating the importance of individual frequency components to the perceived loudness of multitone complexes (Jesteadt et al., 2017; Leibold & Jesteadt, 2007; Oberfeld et al., 2012). By using a method called perceptual weight analysis, these studies have typically presented sound ranges with constant overall loudness but random trial-by-trial frequency level variation, and subsequently obtained weights by computing the correlation between these variations and the responses (Joshi et al., 2016). Consistently, these studies have shown that higher weights are given to the lower and the higher frequencies than to the middle frequencies, indicating a more substantial contribution of the edges to the overall loudness of a complex. Moreover, similar to the conclusions of our second experiment, the increased saliency of the edges seems to depend not on the absolute value of these frequencies, but rather on their relative position in a complex.

Beyond the auditory domain, contrasting and analogous effects emerge in the visual domain (for reviews, see Carrasco, 2011, 2018). Unlike the observed facilitated recognition of melodies at the edges of the acoustic scene, spatial resolution in the visual domain adheres to a contrasting hierarchy, wherein central objects are identified more accurately and quickly, and in more detail than objects in the periphery or edges (Cannon, 1985; Rijsdijk et al., 1980). A decisive influence of attention has been well reported that allows for perceptual higher resolutions and thus the recognition of finer details at attended locations (Dosher & Lu, 2000; Huang & Dobkins, 2005; Lee et al., 1997). Moreover, directing attention through prior cues has been shown to compensate for the preferential focus on central objects in the visual scene, enabling objects at the periphery to be brought into focus (Carrasco & Yeshurun, 1998). This parallels observations in the acoustical realm (Alain & Arnott, 2000; Bey & McAdams, 2002) and mirrors our results, where auditory cues could be leveraged to spotlight specific frequency regions.

In conclusion, the results of our study suggest the absence of distinct biases towards absolute frequency regions. Instead, we observed a relative frequency bias with a specific salience of edge frequencies in auditory pattern matching. Future work should probe the generality of these findings and the ways in which they extrapolate to naturalistic acoustic scenes.

## Supplementary information

Below is the link to the electronic supplementary material.

Supplementary file1 (DOCX 377 KB)

## Data Availability

All experimental data can be downloaded under the following link: https://github.com/Music-Perception-and-Processing/SpectralPreferences

## Code availability

All analysis scripts can be downloaded under the following link: https://github.com/Music-Perception-and-Processing/SpectralPreferences
