# Supplementary material for: Enhanced salience of edge frequencies in auditory pattern recognition
Source: Atten Percept Psychophys. 2024 Oct 26;86(8):2811–20. doi: 10.3758/s13414-024-02971-x (PMC11652571; doi:10.3758/s13414-024-02971-x)
Supplement: Supplementary file 1 — Supplementary file1 (DOCX 377 KB) [file 13414_2024_2971_MOESM1_ESM.docx]

**Enhanced salience of edge frequencies in auditory pattern recognition - Supplementary Material**

**Table 1:** d’ scores for Experiment 1

Average d’ scores in Experiment 1. Scores for the detection of melodies are listed by the frequency band in which they occurred and presentation order. Isolated target melody cues were either presented before the mixture of melodies (“Target-Mix”) or after the mixture (“Mix-Target”). The square brackets contain 95% confidence intervals.

|  | 65 Hz | 215 Hz | 441 Hz | 783 Hz | 1230 Hz | 2080 Hz |  |
| --- | --- | --- | --- | --- | --- | --- | --- |
| Target-Mix | 2.93  [2.63 - 3.17] | 2.68  [2.39 - 2.93] | 2.68  [2.28 – 3.00] | 2.67  [2.30 - 2.99] | 2.16  [1.78 - 2.57] | 2.96  [2.72 - 3.17] | |
| Mix-Target | 2.21  [1.77 - 2.65] | 1.24  [0.84 - 1.70] | 1.50  [1.12 - 1.87] | 1.32  [0.90 - 1.72] | 1.20  [0.83 - 1.68] | 2.04  [1.61 - 2.46] | |

**Table 2:** d’ scores for Experiment 2

Average d’ scores in Experiment 2. Scores for the detection of melodies are listed by the frequency band in which they occurred, frequency region and presentation order. The mixtures in Experiment 2 contained melodies either in the first four frequency regions of Experiment 1 (“Low Region”) or last four frequency regions (High Region). Isolated target melody cues were either presented before the mixture of melodies (“Target-Mix”) or after the mixture (“Mix-Target”). The square brackets contain 95% confidence intervals.

|  |  | Frequency band1 | Frequency band2 | Frequency  band3 | Frequency  band4 |
| --- | --- | --- | --- | --- | --- |
| Low Region  (65 - 783 Hz) | Target-Mix | 2.85  [2.62 - 3.07] | 2.67  [2.46 - 2.88] | 2.45  [2.17 - 2.70] | 3.03  [2.83 - 3.19] |
|  | Mix-Target | 2.93  [2.74 - 3.12] | 2.55  [2.32 - 2.77] | 2.42  [2.19 - 2.67] | 2.93  [2.75 - 3.09] |
| High Region  (441 - 2080 Hz) | Target-Mix | 2.10  [1.74 - 2.47] | 1.72  [1.43 - 2.02] | 1.70  [1.46 - 1.96] | 2.72  [2.45 - 2.95] |
|  | Mix-Target | 2.44  [2.14 - 2.76] | 1.87  [1.54 - 2.17] | 1.44  [1.13 - 1.77] | 2.26  [2.01 - 2.54] |

**Supplementary Figure 1.** Alternative analysis of detection accuracy in Experiment 1. Accuracy is represented as d’ scores. The square, circle, diamond and star marks denote the mean scores for melodies in the specified frequency bands. The square and diamond marks indicate the presentation order "TM" where the target cue was presented first followed by a mixture. The circle and star marks indicate the presentation order "MT," where a mixture was presented first followed by the target cue. The circle and square marks represent the detection in musical scenes where a frequency band adjacent to the target was muted. The diamond and star marks represent the detection in musical scenes where a frequency band distant from the target was muted. Error bars represent 95% CIs computed using bootstrapping.


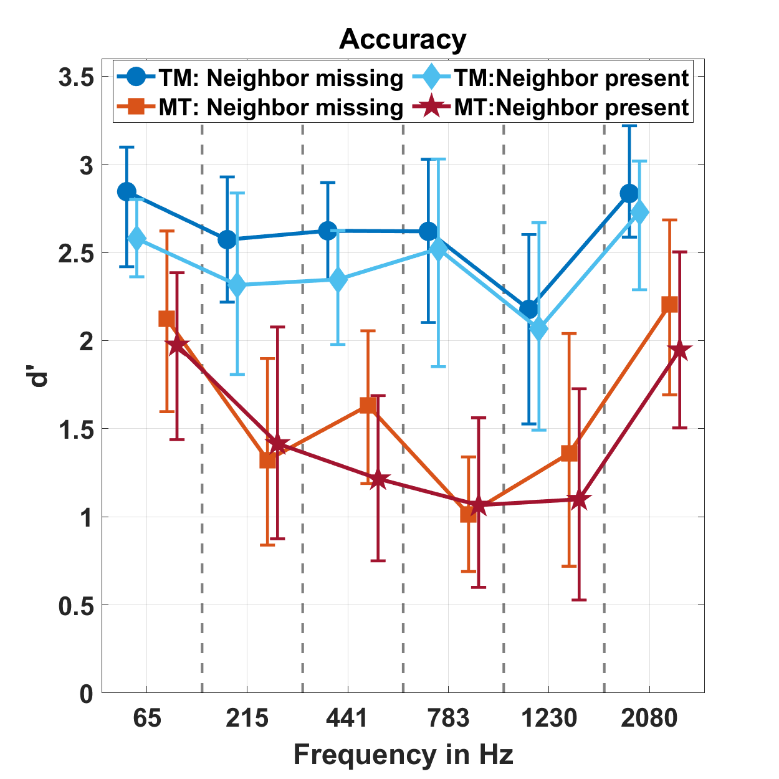


**Experiment 1 – Presence of neighbor**


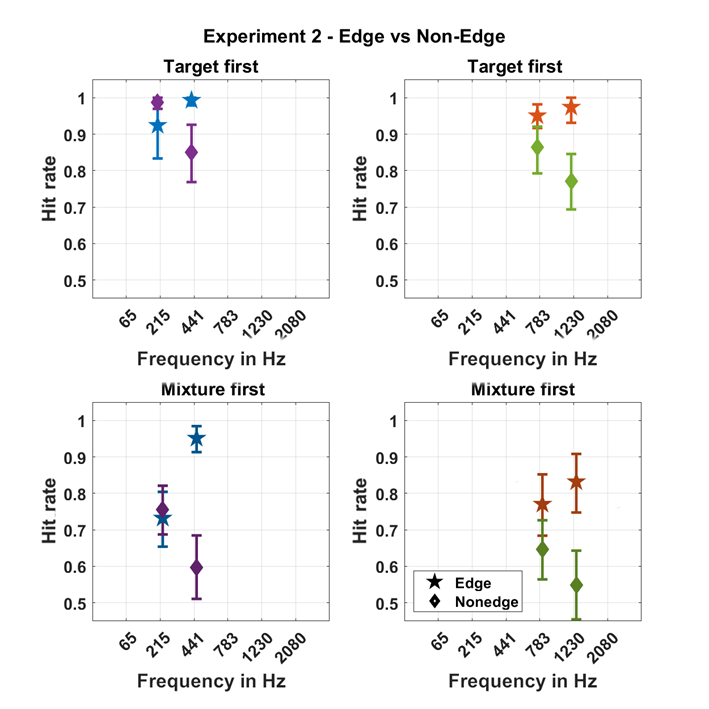


**Experiment 2 – Edge vs Non-Edge**

**Supplementary Figure 2.** Differences between detection accuracy for melodies that appeared in the center or at the edge of musical mixtures in Experiment 2 represented. Three melodies were presented simultaneously within either a low or high frequency range. The low-frequency range (65 - 783 Hz) is indicated by blue and purple colors, the orange and green represents the high-frequency range (441 - 2080 Hz). The star marks (“Edge”) denote the mean hit rate of target melodies that appeared on the edge of the musical mixture in the specified frequency bands. The circle marks (“Nonedge”) denote the mean hit rate of target melodies that appeared in the center of the musical mixture in the specified frequency bands. Error bars indicate 95% CIs.
